# *P. gingivalis* induces endothelial dysfunction via mitochondrial fission dependent VDAC1-HK2 disassociation

**DOI:** 10.1080/20002297.2026.2643035

**Published:** 2026-03-10

**Authors:** Yi Wang, Shengming Xu, Zichao Zhuang, Congyi Tu, Zhe Zhou, Tianhao Chen, Mengting Wu, Bin Lu, Pengcheng Ye, Xia Fan, Rongdang Hu, Hui Deng

**Affiliations:** a Institute of Stomatology, School and Hospital of Stomatology, Wenzhou Medical University, Wenzhou, Zhejiang, People's Republic of China; b Department of Orthodontics, School and Hospital of Stomatology, Wenzhou Medical University, Wenzhou, Zhejiang, People's Republic of China; c Department of Periodontology, School and Hospital of Stomatology, Wenzhou Medical University, Wenzhou, Zhejiang, People's Republic of China

**Keywords:** Endothelial dysfunction, mitochondria, mPTP, Drp1, *Porphyromonas gingivalis*

## Abstract

**Background:**

Mitochondrial dysfunction contributes to *Porphyromonas gingivalis* (*P. gingivalis*)*-*impaired endothelial function. Given the critical role of the mitochondrial permeability transition pore (mPTP) in mitochondrial homeostasis, this study explored how *P. gingivalis* promotes dynamin-related protein 1 (Drp1)–dependent mPTP overactivation, leading to mitochondrial damage and endothelial dysfunction.

**Materials and methods:**

Mitochondrial and endothelial functions were evaluated in *P. gingivalis*–infected human aortic endothelial cells (HAECs) and C57BL/6 mice. Western blotting, immunofluorescence, and co-immunoprecipitation were used to assess the mitochondrial dynamics and mPTP-related protein interactions. Aortic vasodilation and endothelial integrity were examined following treatment with the Drp1 inhibitor Mdivi-1 or mPTP inhibitor cyclosporin A (CsA).

**Results:**

*P. gingivalis* infection induced significant mitochondrial fragmentation, excessive mPTP opening, and impaired endothelium-dependent vasorelaxation. These changes were associated with enhanced *p*-Drp1 and its translocation to mitochondria. Mechanistically, *P. gingivalis* promoted voltage-dependent anion channel 1 (VDAC1) oligomerization in the out membrane of mitochondrial via *p*-Drp1 activation, which in turn disrupted the VDAC1–hexokinase 2 (HK2) interaction, facilitating mPTP opening. Inhibition of Drp1 and mPTP opening significantly alleviated mitochondrial dysfunction and restored endothelial function both in vitro and in vivo.

**Conclusion:**

*P. gingivalis* impairs endothelial function via Drp1–VDAC1–HK2–mediated mPTP overactivation, highlighting a potential therapeutic target against vascular injury in periodontal infection.

## Key finding

This study found that *P. gingivalis* induces endothelial dysfunction through Drp1-VDAC1-HK2 mediated mPTP overactivation.

## Introduction

The vascular endothelium plays a central role in maintaining cardiovascular homeostasis by regulating redox balance, vasodilation and barrier function [1]. Disruption of endothelial integrity is a critical early event in the pathogenesis of atherosclerosis and other cardiovascular diseases [2]. While traditional risk factors such as hypertension, dyslipidemia, and smoking are well-established, increasing attention has turned to microbial insults, especially those arising from chronic infections, as potential contributors to endothelial dysfunction [2]. Certain pathogens have been shown to enter the bloodstream, triggering oxidative stress and mitochondrial injury in endothelial cells, but the precise molecular mechanisms remain incompletely understood [3]. Among these pathogens, *Porphyromonas gingivalis* (*P. gingivalis*), the keystone bacterium in periodontitis, has been identified in atherosclerotic plaques [4], and experimental evidence demonstrates that it can adhere to and invade vascular endothelial cells [5–7], supporting a potential causal effect. Interestingly, our earlier study showed that *P. gingivalis* enables its persistence within endothelial cells by impairing lysosomal function [8]. Once internalized, *P. gingivalis* has been shown to damage endothelial function by promoting oxidative stress and inflammation [6]. Consequently, understanding how *P. gingivalis* induces endothelial dysfunction may help elucidate the mechanistic link between periodontitis and atherosclerosis.

Mitochondria are central to cellular energy metabolism and redox regulation, and their dysfunction can disrupt endothelial cell function and vasomotor activity, contributing to vascular pathology [9]. Previous studies have suggested that *P. gingivalis* disrupts mitochondrial function in endothelial cells is related to dynamin-related protein 1 (Drp1)-regulated mitochondrial fission [10]. Our study also showed that *P. gingivalis* impaired endothelial function is closely related to Sirt3-dependet mitochondrial function [11]. Yet, the molecular mechanisms underlying mitochondrial dynamics in *P. gingivalis* induced endothelial function warrants further elucidation.

Drp1, known as the key regulator of mitochondrial fission, could also modulate mitochondrial permeability through its interaction with the mitochondrial permeability transition pore (mPTP), a channel spanning both mitochondrial membranes that plays a key role in controlling membrane permeability [12]. Aberrant mPTP opening has been closely linked to mitochondrial injury and vascular endothelial dysfunction. In hypertensive mice, excessive mPTP activation impairs nitric oxide (NO)–mediated vasodilation in hypertensive mice, highlighting its potential role in vascular disease progression [13]. Importantly, the regulation of mPTP is influenced by outer mitochondrial membrane proteins such as voltage-dependent anion channel 1 (VDAC1) and hexokinase 2 (HK2), which are thought to stabilize membrane structure through protein-protein interactions [14–16]. However, whether the VDAC1–HK2 complex contributes to *P. gingivalis* induced mitochondrial dysfunction remains unknown.

In this study, we performed both in vitro and in vivo experiments to examine how *P. gingivalis* infection affects mitochondrial integrity and endothelial function, with a particular focus on the regulatory role of the Drp1–mPTP axis. And we further investigated whether the VDAC1–HK2 complex mechanistically contributes to Drp1-dependent mPTP opening in response to *P. gingivalis*. By delineating these pathways, our study seeks to uncover the molecular mechanisms by which periodontal infection contributes to endothelial dysfunction, thereby advancing our understanding of the biological basis linking periodontitis to cardiovascular disease.

## Materials and methods

### Cell culture and reagents

Human aortic endothelial cells (HAECs; iCell Bioscience, China) were cultured in Endothelial Cell Medium (ScienCell, USA) supplemented with 5% fetal bovine serum and 1% endothelial cell growth supplement. Cells were maintained at 37 °C in a humidified 5% CO₂ incubator, and experiments were performed using passages 5–8. Mdivi-1, the mPTP inhibitor cyclosporin A (CsA), and the VDAC1 oligomerization inhibitor VBIT-4 were purchased from TargetMol (USA).

### Bacterial culture

The *P. gingivalis* strains W83 and 33277 (ATCC) were initially cultivated on blood agar plates containing 10% defibrinated sheep blood (Solarbio, China) for 5–7 days at 37 °C under strictly anaerobic conditions. Bacteria were then transferred to brain heart infusion broth (Solarbio, China) supplemented with hemin (5 μg/mL) and vitamin K (5 μg/mL) and maintained for more than 24 hours in an anaerobic environment at 37 °C. Unless otherwise specified, *P. gingivalis* strain W83 was used for all experiments presented in the main figures. The strain ATCC 33277 was employed for validation experiments as indicated in the Supplementary Information.

### Animal procedures

All animal procedures were conducted in accordance with the ARRIVE guidelines and approved by the Institutional Animal Care and Use Committee of Wenzhou Medical University (xmsq2023-0927). Twenty-four six-week-old male C57BL/6J mice (SPF Biotechnology Co., Ltd., China) were acclimatized for one week and then randomly assigned to 1) Uninfected group (*n* = 6) received topical application of 2% carboxymethyl cellulose sodium (CMC) (Solarbio, China) to the buccal mucosa of the second maxillary molars bilaterally (0.1 mL per side), five times per week for six weeks; 2) *P. gingivalis* group treated with a suspension of *P. gingivalis* (1 × 10⁸ CFU/mL) prepared in 2% CMC using the same dosing regimen; 3) *P. gingivalis* + CsA group, receiving *P. gingivalis* plus intraperitoneal CsA (10 mg/kg, once weekly); 4) *P. gingivalis +* Mdivi-1 group, receiving *P. gingivalis* plus intraperitoneal Mdivi-1 (20 mg/kg, once weekly). The oral inoculation procedure for *P. gingivalis* was based on previously reported methods, with minor modifications [17–19]. Additional methodological details are provided in the Supplementary Materials.

### Western blot analysis

Cellular proteins were extracted using RIPA buffer (TargetMol, USA) containing protease inhibitors. Protein concentrations were measured with a BCA kit (Beyotime, China). Equal protein amounts were separated by SDS–PAGE and transferred to PVDF membranes. After blocking for 10 min with Protein-Free Rapid Blocking Buffer (Epizyme, China), membranes were incubated overnight with primary antibodies (listed in Supplementary Table S1), followed by 1 h incubation with HRP-conjugated secondary antibodies. Signals were visualized using BeyoECL Plus (Beyotime, China) and quantified by densitometry in ImageJ (v1.54f; NIH, USA).

### VDAC1 cross-linking assay

VDAC1 oligomerization was assessed by cross-linking with 0.5 mM ethylene glycol bis (succinimide succinate) (EGS) in PBS (pH 7.4) for 30 min, quenched with 20 mM Tris-HCl (pH 7.8), and analyzed by SDS–PAGE and immunoblotting with anti-VDAC1 antibody (Abcam, UK).

### Aortic ring immunofluorescence staining and IHC

For immunofluorescence staining, 5-µm paraffin or frozen sections were prepared and incubated with a primary antibody, followed by either Cy3-conjugated Goat Anti-Mouse IgG or Alexa Fluor 488-conjugated Goat Anti-Mouse IgG (Beyotime, China). Nuclei were counterstained with DAPI (Beyotime, China), and images were captured using a Leica laser scanning confocal microscope (Germany).

### Organ bath assay for vasorelaxation evaluation

Thoracic aortic rings were mounted in organ baths with oxygenated Krebs–Henseleit solution (37 °C, 95% O₂/5% CO₂) and connected to a physiological recording system (RM6240, Chengdu Instrument Factory, China). After 5 mN resting tension and 60 min equilibration, vascular reactivity was assessed by (1) high-K⁺ (60 mM) contraction, (2) norepinephrine (1 µM) pre-constriction followed by cumulative acetylcholine (Ach, 10^−9^–10^−5^ M), and (3) repeated pre-constriction followed by sodium nitroprusside (SNP, 10^−9^–10^−5^ M). More details are described in Supplementary Materials.

### Statistical analyses

Normality of data distribution was assessed using the Shapiro–Wilk test, and all datasets met the assumption of normality. Results are expressed as mean ± SEM. Comparisons between two groups were performed using Student’s *t*-test, while one-way ANOVA with Tukey’s post hoc test was applied for multiple comparisons. Aortic vasorelaxation data were evaluated by two-way ANOVA followed by Bonferroni’s post hoc test. Statistical analyses were conducted with GraphPad Prism 5.0 (GraphPad, La Jolla, CA), and *P* < 0.05 was considered significant.

**Further methodological details, including additional experimental procedures, can be found in the Supplementary Materials.**

## Results

### *P. gingivalis* enhances Drp1 phosphorylation and its mitochondrial translocation in HAECs

**Figure 1.**
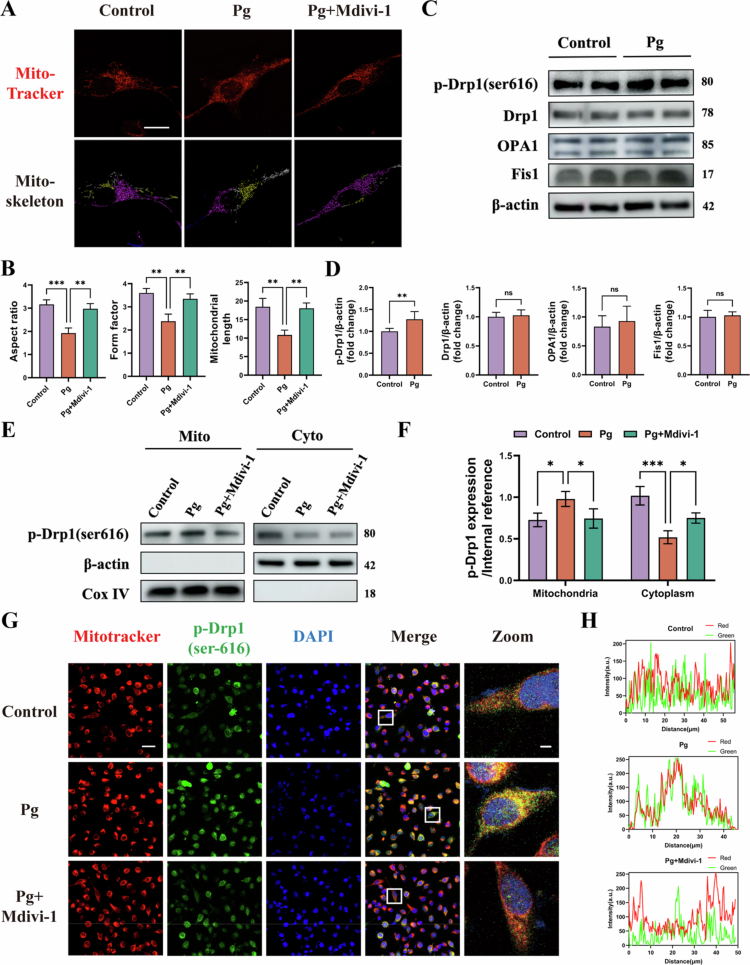
*P. gingivalis* promotes Drp1 phosphorylation and mitochondrial translocation in HAECs. (A, B) Representative confocal images of mitochondrial morphology in *P. gingivalis*-infected HAECs (MOI = 100 for 24 h) following Mdivi-1 (50 μM) treatment. Mitochondrial length in each group was calculated by Image J. Scale bars =10 μm. (*n* = 3). (C, D) *p*-Drp1(ser616), Drp1, OPA1, Fis1 protein levels in HAECs at 24 h after *P. gingivalis* infection (MOI = 100) and quantitative bar chart. (*n* = 3). (E, F) Relative protein expression of *p*-Drp1(ser616) in cytoplasmic and mitochondrial fractions of HAECs and quantitative bar chart. (*n* = 3). (G, H) Representative immunofluorescence images and statistics analysis showing the co-location of mitochondria (red) and *p*-Drp1 (green) in HAECs. Scale bars: 20 μm (original) and 2 μm (Zoom). Pg, *P. gingivalis*. All numbers (*n*) are biologically independent experiments. ns = not significant. *P < 0.05. **P < 0.01. ***P < 0.001.

Micro-CT images of the maxillae demonstrated that periodontal destruction was induced by oral application of *P. gingivalis* (Supplementary Figure S1A & B). We then examined whether orally administrated *P. gingivalis* could translocate to aortic tissues. FISH revealed *P. gingivalis* signals in aortic tissues (Supplementary Figure S1C), indicating direct interaction with vascular endothelium. Given the critical role of mitochondria in cellular function, we assessed mitochondrial morphology in infected HAECs. *P. gingivalis* markedly induced mitochondrial fragmentation, reducing mean mitochondrial length by nearly 38% compared with controls (*P* < 0.05) (Figure 1A, B). This observation indicated that *P. gingivalis* infection may alter mitochondrial dynamics, which led us to further investigate the expression of key proteins regulating this process. Consequently, we found that phosphorylation of Drp1 at Ser616 was markedly increased (Figure 1C, D), and this effect was consistent across *P. gingivalis* strains (Supplementary Figure S2A & B). Subcellular fractionation showed increased mitochondrial and decreased cytosolic *p*-Drp1 (Ser616) (Figure 1E, F), consistent with its mitochondrial translocation [20]. Immunofluorescence confirmed enhanced co-localization of *p*-Drp1(Ser616) with mitochondria (Figure 1G, H). Furthermore, Mdivi-1, a specific Drp1 inhibitor, restored normal mitochondrial morphology and prevented *p*-Drp1 mitochondrial translocation (Figure 1A, E–H). Consistently, genetic silencing of Drp1 via siRNA also rescued the mitochondrial network from *P. gingivalis*-induced fragmentation (Supplementary Figure S2C - E), collectively indicating that *P. gingivalis*-induced mitochondrial fission is dependent on Drp1 activation.

### Drp-1 inhibition alleviates *P. gingivalis*-induced mitochondrial and endothelial dysfunction

**Figure 2.**
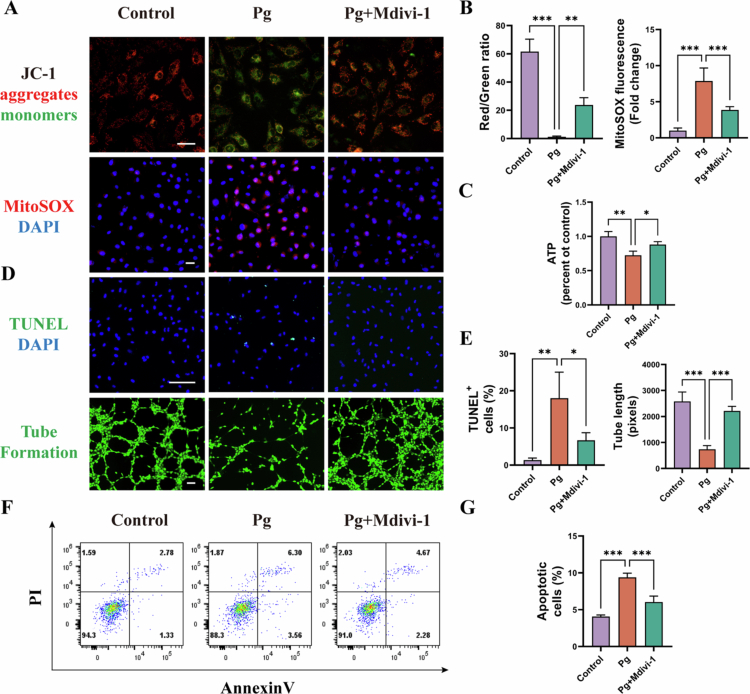
Mdivi-1 rescues *P. gingivalis*-induced mitochondrial and endothelial dysfunction. HAECs was pretreated with or without Mdivi-1 (50 μM) and then infected with *P. gingivalis* for 24 h (MOI = 100). (A, B) JC-1, MitoSOX staining assay and quantitative bar chart. Scale bars = 20 μm. (*n* = 3). (C) ATP production in HAECs. (*n* = 3). (D, E) TUNEL staining, tube formation assay and quantitative bar chart. Scale bars = 200 μm. (*n* = 3). (F, G) Apoptotic ratio in HAECs with quantitative data. (*n* = 3). Pg, *P. gingivalis*. All numbers (*n*) are biologically independent experiments. *P < 0.05. **P < 0.01. ***P < 0.001.

Mitochondrial dynamics are intrinsically linked to mitochondrial homeostasis. In HAECs, *P. gingivalis* infection significantly reduced mitochondrial membrane potential, as evidenced by a decrease in JC-1 red/green fluorescence ratio (*P* < 0.001), increased mtROS production 7.8-fold (*P* < 0.001), and lowered ATP generation by 27.5% (*P* < 0.01) (Figure 2A–C). It also increased TUNEL-positive cells and nearly doubled the proportion of apoptotic cells (*P* < 0.001) (Figure 2D–G). Endothelial tube formation was markedly impaired, with a 71.4% reduction in total tube length (*P* < 0.001) (Figure 2E). Notably, inhibition of Drp1 by Mdivi-1 significantly restored mitochondrial function and rescued endothelial dysfunction (Figure 2A–G).

### Drp1-mediated mPTP opening contributes to *P. gingivalis*-induced mitochondrial and endothelial dysfunction

**Figure 3.**
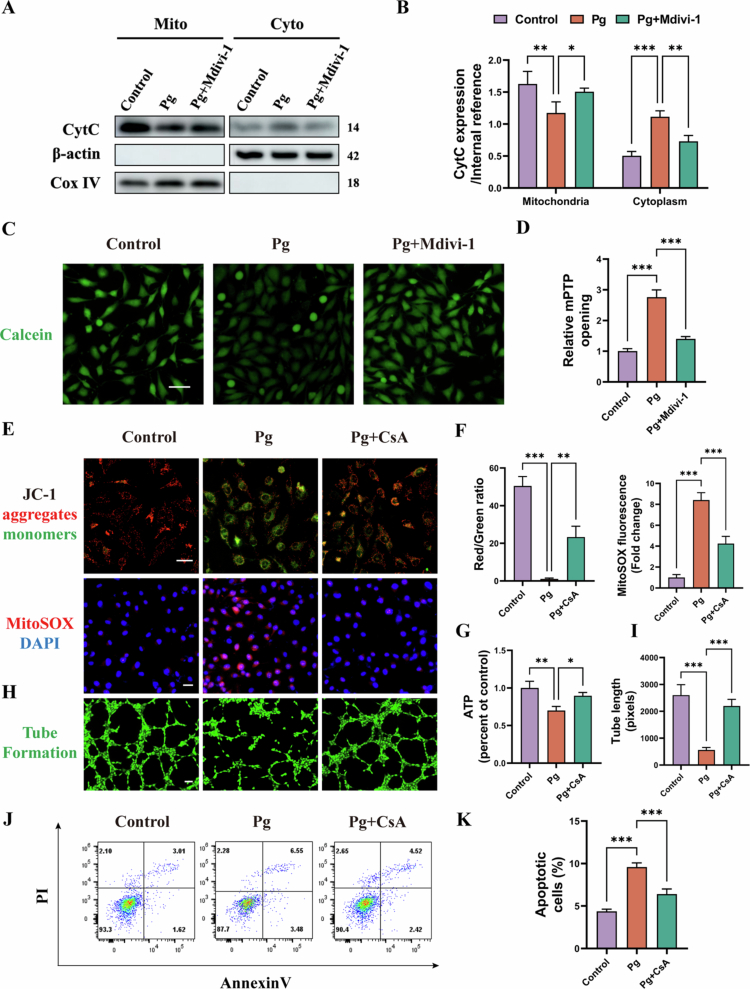
*P. gingivalis* facilitates Drp1-dependent mPTP opening in endothelial cells. HAECs was pretreated with or without Mdivi-1 (50 μM) and then infected with *P. gingivalis* for 24 h (MOI = 100). (A, B) Representative immunoblots and quantification of CytC levels in cytosolic and mitochondrial fractions of HAECs *p*. (*n* = 3). (C, D) Calcein staining assay and quantitative bar chart. Scale bars = 20 μm. (*n* = 3). HAECs was pretreated with or without CsA (1 μM) and then infected with *P. gingivalis* for 24 h (MOI = 100). (E, F) JC-1, MitoSOX staining assay and quantitative bar chart. Scale bars = 20 μm. (*n* = 3). (G) ATP production in HAECs. (*n* = 3). (H, I) Tube formation assay and quantitative bar chart. Scale bars = 200 μm. (*n* = 3). (J, K) Apoptotic ratio in HAECs with quantitative data. (*n* = 3). Pg, *P. gingivalis*. All numbers (*n*) are biologically independent experiments. *P < 0.05. **P < 0.01. ***P < 0.001.

Our study showed *P. gingivalis* infection increased mPTP opening, as indicated by CytC translocation from mitochondria to cytosol and calcein quenching (*P* < 0.001) (green) (Figure 3A–D). Importantly, this effect was reversed by Mdivi-1 and further confirmed by the genetic silencing of Drp1, which similarly attenuated *P. gingivalis*–induced mPTP over-opening (Figure 3C, D, Supplementary Figure S2F & G), identifying Drp1 as a critical mediator of this process. Moreover, inhibition of mPTP with CsA restored mitochondrial membrane potential, ATP production, and tube formation, while reducing mtROS levels by nearly 50% and the proportion of apoptotic cells by about 30% (Figure 3E–K). Collectively, these results suggested that Drp1-mediated mPTP opening plays a pivotal role in *P. gingivalis*-induced mitochondrial and endothelial dysfunction.

### Drp1-dependent VDAC1 oligomerization is required for *P. gingivalis* induced mitochondrial dysfunction

**Figure 4.**
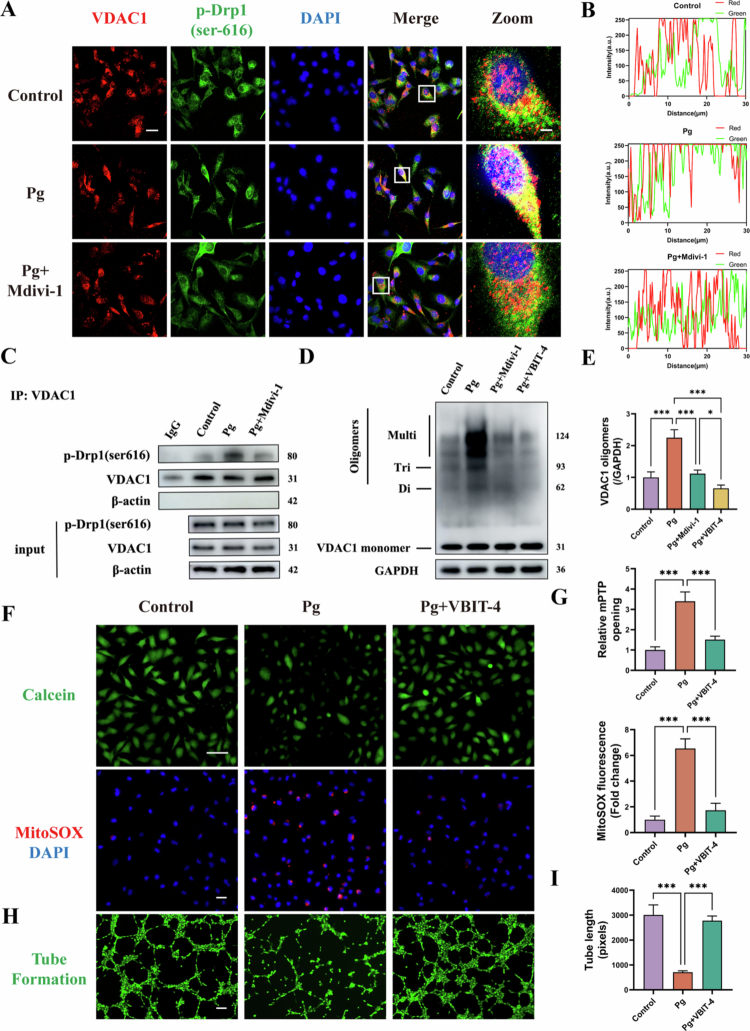
*Drp1-dependent VDAC1 oligomerization is required for P. gingivalis induced mitochondrial dysfunction.* HAECs was pretreated with or without Mdivi-1 (50 μM) and then infected with *P. gingivalis* for 24 h (MOI = 100). (A, B) Representative immunofluorescence images and statistics analysis showing the co-location of VDAC1 (red) and *p*-Drp1 (green) in HAECs. Scale bars: 20 μm (original) and 2 μm (Zoom). (C) The interaction of *p*-Drp1 and VDAC1 was validated using Co-immunoprecipitation assays. (*n* = 3). (D, E) Immunoblot of VDAC1 cross-linking in HAECs pretreated with Mdivi-1 (50 μM) or VBIT-4 (20 μM) and quantitative analysis of oligomers. (*n* = 3). (F, G) Calcein, MitoSOX staining assay and quantitative bar chart. Scale bars = 20 μm. (*n* = 3) (H, I) Tube formation assay and quantitative bar chart. Scale bars = 200 μm. (*n* = 3). Pg, *P. gingivalis*. All numbers (*n*) are biologically independent experiments. *P < 0.05. ***P < 0.001.

Our previous findings demonstrated that *P. gingivalis* promotes mitochondrial translocation of *p*-Drp1. To further clarify its role in mPTP opening, we examined its interaction with VDAC1, a key outer mitochondrial membrane protein. Immunofluorescence showed increased VDAC1-*p*-Drp1 colocalization in *P. gingivalis*-infected HAECs, suggesting enhanced binding (Figure 4A, B). Co-immunoprecipitation confirmed this interaction and showed that it could be attenuated by Mdivi-1 treatment (Figure 4C). Additionally, cross-linking immunoblotting showed that *P. gingivalis* increased VDAC1 oligomer formation. This effect was abrogated by Drp1 inhibition and similarly suppressed by the VDAC1 oligomerization inhibitor VBIT-4 (Figure 4D, E). Together, these findings indicated that *P. gingivalis* drives VDAC1 oligomerization through Drp1.

Furthermore, we used VBIT-4 and found that blocking VDAC1 oligomerization attenuated *P. gingivalis*–induced mPTP overactivation and mtROS generation while restoring endothelial tube formation (Figure 4F–I), implying that *P. gingivalis*–induced mitochondrial and endothelial dysfunction is mediated by VDAC1 oligomerization.

### *P. gingivalis* promotes the dissociation of HK2 from VDAC1

**Figure 5.**
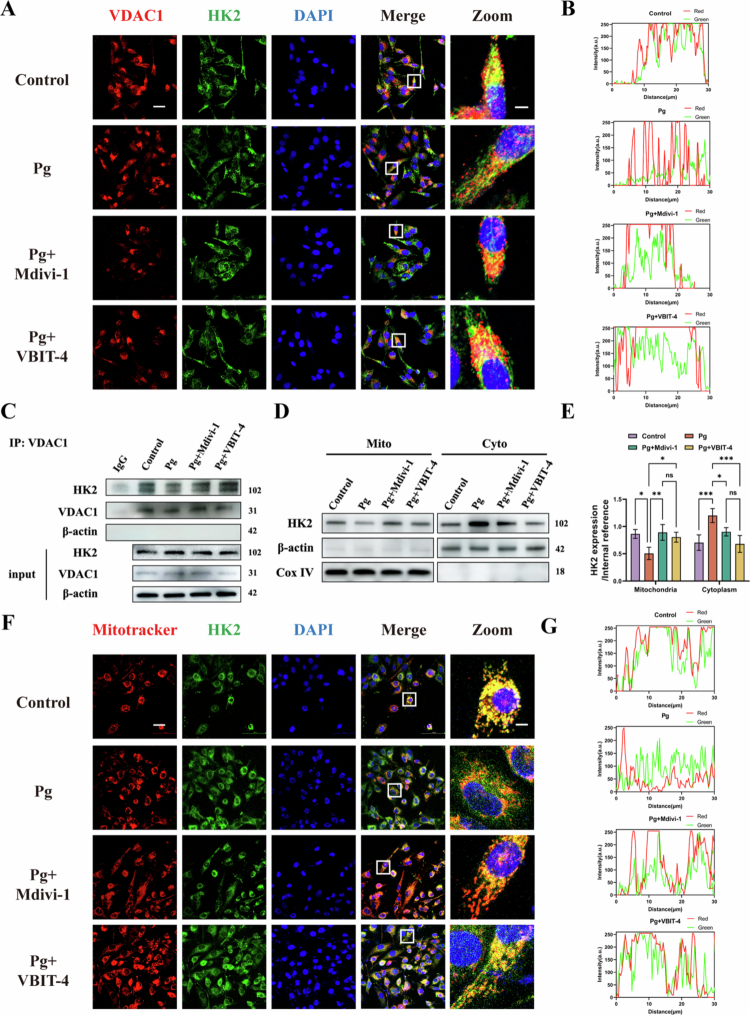
*P. gingivalis* triggers HK2 dissociation from VDAC1. HAECs were pretreated with Mdivi-1 (50 μM) or VBIT-4 (20 μM), followed by infection with *P. gingivalis* for 24 h. (MOI = 100). (A, B) Representative immunofluorescence images and statistics analysis showing the co-location of VDAC1 (red) and HK2 (green) in HAECs. Scale bars: 20 μm (original) and 5 μm (Zoom). (C) The interaction of VDAC1 and HK2 was validated using Co-immunoprecipitation assays. (*n* = 3). (D, E) Representative immunoblots and quantification of HK2 levels in cytosolic and mitochondrial fractions of HAECs (*n* = 3). (F, G) Representative immunofluorescence images and statistics analysis showing the co-location of mitochondria (red) and HK2 (green) in HAECs. Scale bars: 20 μm (original) and 5 μm (Zoom). (*n* = 3). Pg, *P. gingivalis*. All numbers (*n*) are biologically independent experiments. ns = not significant. *P < 0.05. **P < 0.01. ***P < 0.001.

The VDAC1–HK2 interaction is critical for regulating mPTP opening [21]. Consistent with this role, computational modeling identified multiple putative binding sites between VDAC1 and HK2 (Supplementary Figure S2H). Upon *P. gingivalis* infection, the colocalization between VDAC1 and HK2 was diminished, suggesting the dissociation of HK2 from VDAC1. Notably, the restoration of VDAC1–HK2 binding was achieved by pharmacological inhibition of Drp1 (Mdivi-1) or VDAC1 oligomerization (VBIT-4), as well as genetic silencing of Drp1 (Figure 5A–B), a result corroborated by co-immunoprecipitation (Figure 5C, Supplementary Figure S2I). In line with VDAC1’s outer-membrane localization, subcellular fractionation revealed a shift of HK2 from mitochondria to cytosol after infection, indicating mitochondrial HK2 loss (Figure 5D–E). Immunofluorescence further confirmed reduced HK2–mitochondria colocalization in infected cells, which was rescued by Mdivi-1 and VBIT-4 (Figure 5F–G). Collectively, these data indicated that *P. gingivalis* could promote VDAC1 oligomerization, thereby disrupting the VDAC1–HK2 complex.

### *P. gingivalis* induces endothelial dysfunction in mice through Drp1-mediated opening of mPTP

**Figure 6.**
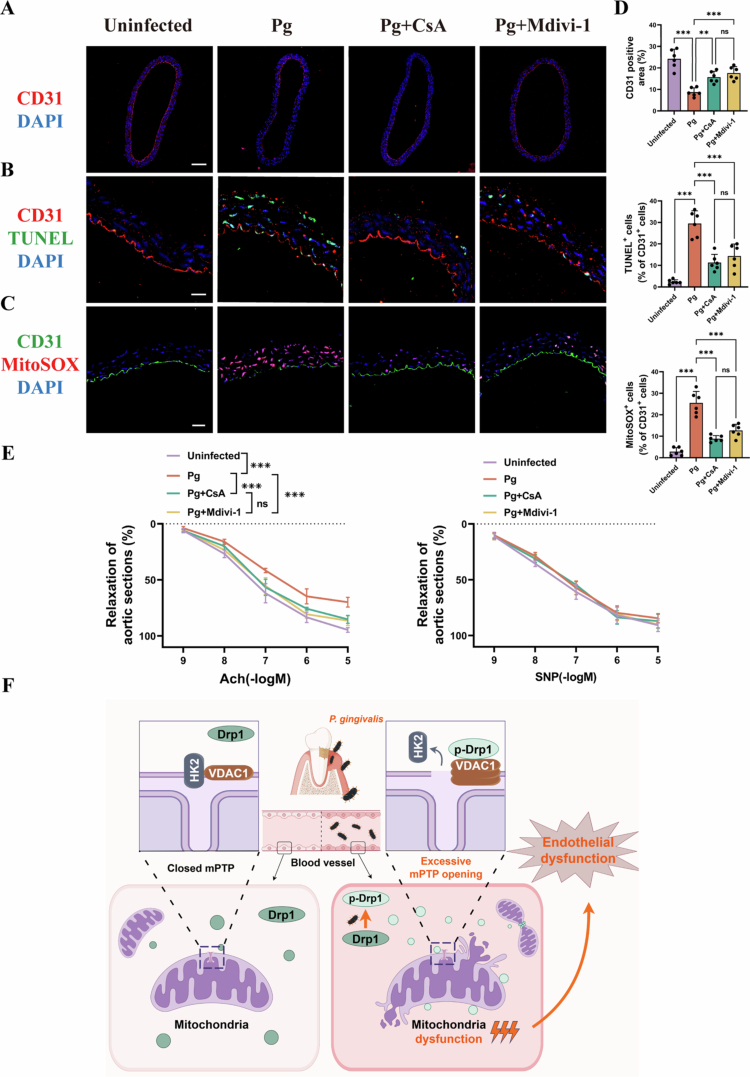
Drp1-mediated mPTP opening contributes to *P. gingivalis*-induced endothelial dysfunction in vivo. (A) CD31 immunofluorescence of aorta tissue sections, scale bars = 100 μm. (*n* = 6). (B) TUNEL stained aorta tissue sections, scale bars = 20 μm. (*n* = 6). (C) MitoSOX stained aorta tissue sections, scale bars = 20 μm. (*n* = 6). (D) Quantitative bar chartof (A)–(C). (E) Concentration–response curves of mice aortic rings after exposure to Ach or SNP treatments, from Vehicle-treated mice, *P. gingivalis*-infected mice, CsA (20 mg/kg) treated *P. gingivalis* infected mice and Mdivi-1 (30 mg/kg) treated *P. gingivalis* infected mice aorta tissue sections. (*n* = 3). (F) Proposed molecular mechanism by which *P. gingivalis* induces endothelial dysfunction via Drp1-HK2-mPTP axis. Pg, *P. gingivalis*. All numbers (*n*) are biologically independent experiments. ns = not significant. **P < 0.01. ***P < 0.001.

We next evaluated the effects of *P. gingivalis* infection on endothelial function in vivo. While HE staining revealed no overt structural denudation (Supplementary Figure S3A), immunofluorescence analysis showed a significant reduction in CD31 fluorescence intensity and elevated apoptosis and mtROS levels, indicating enhanced oxidative stress and compromised endothelial integrity (Figure 6A–D). This was accompanied by increased *p*-Drp1 (Ser616) expression and disrupted VDAC1–HK2 co-localization within the endothelial layer (Supplementary Figure S3B & C). Importantly, Mdivi-1 or CsA treatment effectively reversed these alterations (Figure 6A–D).

In organ bath experiments, *P. gingivalis* significantly impaired ACh-induced endothelium-dependent relaxation, which was restored by CsA and Mdivi-1, whereas SNP-induced endothelium-independent relaxation remained unaffected (Figure 6E). These results indicated that *P. gingivalis* impairs endothelium-dependent vasodilation through Drp1-mediated mPTP opening. The molecular hypothesis proposed in this study is illustrated in Figure 6F. After *P. gingivalis* infection, phosphorylated Drp1 expression is upregulated in HAECs, promoting mitochondrial fission. The translocated *p*-Drp1 interacts with VDAC1 on the outer mitochondrial membrane, facilitating VDAC1 oligomerization and disrupting the VDAC1–HK2 association. These events contribute to excessive mPTP opening, ultimately leading to endothelial dysfunction.

## Discussion

Emerging evidence from epidemiological studies and clinical trials demonstrate the strong association between periodontitis and vascular dysfunction [22,23]. Once disseminated into the bloodstream, *P. gingivalis* could invade vascular endothelial cells and impair their function, thereby linking periodontal infection to vascular pathology [24]. However, the molecular basis underlying this connection remains incompletely defined. In this study, we demonstrated that *P. gingivalis* infection triggers marked mitochondrial dysfunction and endothelial impairment through aberrant activation of the Drp1–mPTP axis. Mechanistically, our findings suggest that the dissociation of the VDAC1–HK2 complex are critical upstream events leading to Drp1-dependent mPTP opening. These results provide mechanistic insight into how periodontal pathogens contribute to vascular inflammation and dysfunction, extending the link between oral infection and atherosclerosis.

Endothelial dysfunction is a critical early event in the initiation and progression of atherosclerosis, characterized by impaired vasodilation, oxidative stress, and loss of vascular integrity [25]. In our study, oral inoculation with *P. gingivalis* significantly attenuated acetylcholine-induced, endothelium-dependent vasorelaxation, reflecting reduced nitric oxide (NO) bioavailability and the onset of endothelial dysfunction. This vascular impairment was closely associated with pronounced mitochondrial injury, including elevated mtROS generation, loss of mitochondrial membrane potential, and structural fragmentation. Excessive mitochondrial ROS can directly interfere with the NO signaling pathway, thereby diminishing NO synthesis and bioactivity, while also promoting energy depletion and activating apoptosis-related pathways that compromise endothelial barrier integrity [26]. These mitochondrial alterations collectively account for the reduced vasodilatory capacity and aggravated endothelial injury observed in our model, providing a mechanistic basis for *P. gingivalis*–induced vascular dysfunction. Furthermore, *P. gingivalis* was detected in the aortas of infected mice, indicating that direct microbial insult may contribute to endothelial injury. Our previous work also showed that *P. gingivalis* survives intracellularly within endothelial cells by hijacking mitophagy and impairing lysosomal function [8], supporting its capacity for long-term persistence in vascular environments. In addition to direct bacterial effects, chronic systemic infection induces a persistent, low-grade inflammatory state that further exacerbates oxidative stress, endothelial apoptosis, and vascular dysfunction [27]. Disentangling the relative contributions of direct bacterial invasion and the accompanying inflammatory response to endothelial injury remains challenging and warrants further investigation.

Drp1 is a GTPase that mediates mitochondrial fission and regulates mitochondrial morphology, bioenergetics, and quality control [28]. Hyperactivation of Drp1 leads to excessive fission, mitochondrial depolarization, and mtROS accumulation, events that have been implicated in endothelial dysfunction and atherosclerosis. Previous in vitro studies have shown that *P. gingivalis* induced Drp-1 dependent mitochondrial fission in endothelial cells, contributing to mitochondrial dysfunction and subsequent impaired endothelial function [10,29]. We extend previous findings by demonstrating that inhibition of Drp1 attenuated the *P. gingivalis*-induced impairment of endothelium-dependent vasodilation in mice. In this process, Drp1 inhibition was achieved with Mdivi-1. Rather than lowering protein abundance, Mdivi-1 limits Drp1 GTPase activity and self-assembly [30], providing a pharmacologic means to selectively curb Drp1 activation.

Consistent with our findings, previous studies have shown that Drp1 activation is accompanied by mPTP opening and loss of mitochondrial membrane potential following *P. gingivalis* infection [10,29]. However, these studies did not clarify whether Drp1 directly mediates mPTP opening or acts indirectly as a regulatory factor influencing this process. During mitochondrial fission, activated Drp1 is recruited from the cytosol to the outer mitochondrial membrane, where it forms oligomeric rings that constrict mitochondria [31]. VDAC1, a pore-forming protein on the outer membrane, can serve as one of the docking or coordinating sites that help position Drp1 at fission sites [32]. Importantly, we found that Drp-1 directly interacts with VDAC, which promotes its oligomerization, a structural rearrangement that compromises its binding with HK2. HK2 is traditionally known as a key glycolytic enzyme catalyzing glucose phosphorylation [33]. However, emerging evidence suggested that its association with the mitochondrial outer membrane plays a critical role in regulating mPTP opening [14,34,35]. Under physiological conditions, the HK2–VDAC1 complex maintains mitochondrial membrane potential and limits permeability transition by preventing excessive ion flux across the outer membrane [36]. Once Drp1-induced VDAC1 oligomerization disrupts this complex, HK2 dissociates from mitochondria, resulting in mPTP opening, mitochondrial depolarization, and CytC release.

Interestingly, another hexokinase isoform, HK1, has been implicated in *P. gingivalis* related pathogenesis. It was demonstrated that *P. gingivalis* LPS strengthens the interaction between Drp1 and HK1 macrophages, thereby promoting excessive mPTP opening [28]. This observation aligns well with our findings, further supporting the notion that *P. gingivalis* may modulate the Drp1-mPTP axis through multiple mechanisms to drive abnormal mitochondrial permeability transition. However, whether HK2 dissociation merely reflects a de-inhibition of mPTP or also actively contributes to downstream mitochondrial dysfunction remains to be determined based our current findings. Future investigations are warranted to explore the potential dual role of HK2 dissociation in regulating endothelial mitochondrial homeostasis.

Several limitations have to be addressed. First, although we detected *P. gingivalis* in the aorta, the route by which it escapes ulcerated periodontal epithelium and reaches distant vascular tissues remains unclear. Current evidence suggests that periodontal bacteria may disseminate by ʻhitchhiking’ within circulating host cells, including monocytes [37], erythrocytes [38], and dendritic cells [39]. These cells can internalize viable bacteria and subsequently deliver them to distal vascular endothelium, providing a plausible mechanism for endothelial colonization beyond the oral niche. It should be noted that FISH detects *P. gingivalis* rRNA signals rather than viable bacteria [40], and these signals may partly derive from outer membrane vesicles (OMVs) released by *P. gingivalis* [41]. Verification of bacterial viability therefore requires additional methods such as culture-based assays. Another limitation of this study is that the specific virulence factors responsible for *P. gingivalis*-induced endothelial dysfunction were not investigated. *P. gingivalis* expresses multiple virulence factors, including lysine- and arginine-specific cysteine proteases known as gingipains [42]. Notably, Farrugia et al. showed that *P. gingivalis* disrupt endothelial homeostasis via a gingipain-dependent mechanism [5]. Future studies are needed to determine whether and how these factors directly target Drp1 to induce mitochondrial and endothelial dysfunction. In addition, although we inhibited Drp1 activity using Mdivi-1, genetic approaches are required to exclude potential off-target effects. The use of endothelial-specific Drp1 knockout mice would further clarify the in vivo role of Drp1 in *P. gingivalis*-induced endothelial dysfunction.

Despite certain limitations, this study suggests that Drp1 acts as an upstream regulator of mitochondrial permeability by driving VDAC1 oligomerization and HK2 dissociation. This mechanism links Drp1-mediated mitochondrial fission to mPTP opening and provides a plausible explanation for how *P. gingivalis* infection induces mitochondrial dysfunction and endothelial injury through the Drp1–VDAC1–HK2 axis.

## Conclusions

This study demonstrated that *P. gingivalis* induces endothelial dysfunction via Drp1-mediated mPTP opening through disruption of the VDAC1-HK2 complex. Our findings advanced understanding of the clinically observed link between periodontal infection and atherosclerosis.

## Supplementary Material

Supplementary materials 216.docx Supplementary materials 216.docx

## Data Availability

The data that support the findings of this study are available from the corresponding author upon reasonable request.
