# Supplementary material for: P. gingivalis induces endothelial dysfunction via mitochondrial fission dependent VDAC1-HK2 disassociation
Source: J Oral Microbiol. 2026 Mar 10;18(1):2643035. doi: 10.1080/20002297.2026.2643035 (PMC12981260; doi:10.1080/20002297.2026.2643035)
Supplement: Supplementary materials 216.docx [file ZJOM_A_2643035_SM3069.docx]

***P. gingivalis* induces endothelial dysfunction via mitochondrial fission dependent VDAC1-HK2 disassociation**

Yi Wang^1^, Shengming Xu^1^, Zichao Zhuang^1^, Congyi Tu^1^, Zhe Zhou^1^, Tianhao Chen^1^, Mengting Wu^1^, Bin Lu^1^, Pengcheng Ye^1^, Xia Fan^1^, Rongdang Hu^2^, Hui Deng^3^

1. Institute of Stomatology, School and Hospital of Stomatology, Wenzhou Medical University, Wenzhou, Zhejiang, China

2. Department of Orthodontics, School and Hospital of Stomatology, Wenzhou Medical University, Wenzhou, Zhejiang, China

3. Department of Periodontology, School and Hospital of Stomatology, Wenzhou Medical University, Wenzhou, Zhejiang, China

Correspondences:

Hui Deng, Department of Periodontology, School and Hospital of Stomatology, Wenzhou Medical University, Wenzhou, Zhejiang, China. Email: huideng@wmu.edu.cn

Yi Wang, Institute of Stomatology, School and Hospital of Stomatology, Wenzhou Medical University, Wenzhou, Zhejiang, China. Email: [y.wang@wmu.edu.cn](mailto:y.wang@wmu.edu.cn)

Rongdang Hu, Department of Orthodontics, School and Hospital of Stomatology, Wenzhou Medical University, Wenzhou, Zhejiang, China. Email: rongdanghu@wmu.edu.cn

**Table of Contents**

1. Methods
2. Supplementary Figures
3. Supplementary Table
4. **Methods**

**Animal procedure**

Animal cage positions, treatments, and testing sequences were randomized using a computer-generated number sequence. Mice were allocated to the uninfected, *P. gingivalis*, *P. gingivalis* + CsA, or *P. gingivalis* + Mdivi-1 groups, with each animal assigned a unique identification number. Randomization was performed prior to experimentation to minimize bias. Mice were kept under standard conditions and anesthetized with 2% isoflurane (RWD Life Science, China) before oral and intraperitoneal procedures. Sample size was determined based on preliminary data, published studies, and statistical power analysis (power = 0.8, α = 0.05). To account for potential attrition, six animals were included per group. Upon study completion, animals were euthanized with 5% isoflurane, and aortic tissues were collected for further analysis.

**JC-1 staining, mPTP opening detection and measurement of ATP level**

Mitochondrial function was evaluated through multiple assays. Membrane integrity was determined using JC-1 staining (Beyotime, China), while mPTP opening was assessed with a Mitochondrial Permeability Transition Pore Assay Kit (Beyotime, China) or by detecting Cytochrome c (CytC) distribution between mitochondrial and cytosolic fractions via Western blot. Subcellular fractions were prepared with a Cell Mitochondria Isolation Kit (Beyotime, China). Cellular ATP production was measured using a commercial assay kit (Beyotime, China). All procedures were carried out in accordance with the manufacturers’ instructions.

**MitoSOX assay**

Mitochondrial superoxide production in HAECs was detected using MitoSOX Red (Thermo Fisher Scientific, USA) according to the manufacturer’s protocol. For analysis of aortic endothelial cells, 5-µm cryosections were incubated with 5 µM MitoSOX for 30 min. Fluorescence images were acquired with a fluorescence microscope, and signal intensity was quantified in arbitrary units using ImageJ software (version 1.54f; NIH, Bethesda, MD, USA).

**Apoptosis analysis**

Cell apoptosis was assessed by Annexin V-FITC/PI flow cytometry and TUNEL staining. Flow cytometry was performed using the Annexin V-FITC/PI Apoptosis Kit (APExBIO, USA) according to the manufacturer’s protocol, and samples were analyzed with a flow cytometer (BD Biosciences, USA). For the TUNEL assay, HAECs and aortic rings were fixed, paraffin-embedded, sectioned at 5 µm, and stained with the In Situ Cell Death Detection Kit (Roche, Switzerland).

**siRNA Transfection**

Small interfering RNA (siRNA) targeting Drp1 (si-Drp1) and a non-targeting negative control siRNA (si-NC) were synthesized by TsingKe Biotech (China). siRNA were transfected for 6 hours using lipo3000 (Thermo Fisher Scientific, USA) following the manufacturers’ instructions.

**Tube formation**

The angiogenic capacity was evaluated using a tube formation assay. Briefly, 10 μL of Matrigel (abwbio, China) was dispensed into pre-cooled μ-Slide Angiogenesis dishes (ibidi, Germany) and polymerized at 37 °C for 30 min. HAECs (2.0 × 10⁵ cells/well) were then seeded and cultured for 5 h, followed by staining with Calcein AM (Beyotime, China) for 30 min. Tube structures were visualized under a Zeiss microscope (50×), and total tube length was quantified using ImageJ software.

**Co-immunoprecipitation (Co-IP)**

Cells were lysed with Western/IP lysis buffer (Beyotime, China) supplemented with protease inhibitors (Beyotime, China) and a deacetylase inhibitor cocktail (TargetMol, USA). Following centrifugation, the supernatant was incubated overnight with anti-VDAC1 antibody (Proteintech, China) or control IgG (Beyotime, China). BeyoMag™ Protein A+G Magnetic Beads (Beyotime, China) were added for 8 h, after which the immunoprecipitates were washed and subjected to Western blot analysis.

**Mitotracker staining**

Mitochondria in HAECs were stained with MitoTracker Red CMXRos (Beyotime, China) following the manufacturer’s instructions. Images were acquired using a Leica laser scanning confocal microscope (Germany), and mitochondrial morphology was analyzed with Image-Pro Plus 6.0 software.

**Tissue processing**

Following euthanasia, a 14-15 mm segment of thoracic aorta was excised from each mouse and immediately placed in pre-cooled, oxygenated Krebs–Hensleit (K–H) buffer (Procell, China). For organ bath assays, 3-4 mm vascular rings adjacent to the abdominal aorta were carefully isolated using microdissection scissors.

For histological evaluation, a 6-7 mm portion from the distal thoracic aorta was fixed, embedded in paraffin, and sectioned at 5 μm. Three sections per specimen were analyzed. The remaining aortic tissue was processed for frozen sections: rings were dehydrated, fixed, embedded in optimal cutting temperature compound (Sakura Finetek, USA), and cryosectioned at 5 μm. Three sections from each sample were examined.

**Fluorescence in situ hybridization (FISH)**

FISH on aortic sections was conducted as previously described^1,2^. A *P. gingivalis*-specific oligonucleotide probe (5’-CAATACTCGTATCGCCCG TTATTC-3’) labeled with Cy3 was synthesized by GenePharma (China). Frozen sections were incubated with the probe at 40°C for 12 hours, followed by nuclear counterstaining with DAPI (Beyotime, China). Fluorescent images were captured using a laser scanning confocal microscope (Leica, Germany).

**Micro-computed tomography analysis**

The sagittal orientation of the fixed alveolar bones was scanned using micro-computed tomography (μCT; Bruker SKYSCAN 1276) to evaluate alveolar bone loss (ABL). Scanning parameters were set at 70 kV, 200 μA, and a 300 ms integration time, with a voxel resolution of 10 μm. ABL was quantified as the mean distance between the alveolar bone crest (ABC) and the cementoenamel junction (CEJ) in sagittal sections.

**Organ bath assay for vasorelaxation evaluation**

Vascular reactivity was assessed using a multi-channel physiological signal acquisition and processing system (RM6240, Chengdu Instrument Factory, China) to record changes in isometric tension of aortic rings with high precision. Each ring was mounted between two stainless-steel hooks—one connected to a high-sensitivity tension transducer (range: 0-10 g; resolution: ± 0.1 mN) and the other fixed to the organ bath base. The organ bath was filled with K–H solution maintained at 37 °C and continuously aerated with 95% O_2_/5% CO_2_. Data were sampled at 100 Hz.

Aortic rings were equilibrated under a resting tension of 5 mN (0.5 g) for 1 h. During equilibration, the pre-warmed and oxygenated K–H buffer was refreshed every 20 min, and baseline tension was readjusted every 5 min to compensate for stress relaxation, maintaining fluctuations within ≤5%. Contractile and relaxation responses were then assessed as follows:

1. Rings were stimulated with 7 mL of high-K⁺ K-H solution (60 mM), and maximal contraction was recorded upon reaching a steady-state plateau.
2. After three rinses with pre-warmed K-H solution and baseline recovery, the high-K⁺ stimulation was repeated to confirm reproducibility.
3. Following washout and a 15 min equilibration, rings were contracted with 1 μM norepinephrine (NE) until a stable plateau was achieved.
4. Endothelium-dependent relaxation was evaluated by cumulative addition of acetylcholine (ACh, 10^-9^-10^-5^ M). Each concentration was added only after the previous response reached a stable plateau.
5. After washout and re-equilibration, NE-induced precontraction was repeated, and endothelium-independent relaxation was assessed using cumulative concentrations of sodium nitroprusside (SNP, 10^-9^-10^-5^ M) following the same protocol.

Vasodilatory responses were continuously recorded, and relaxation was expressed as a percentage of the NE-induced precontraction:

$$\text{Relaxation (\%)}=\frac{\text{Tension reduction (ACh or SNP-induced)}}{\text{Tension induced by NE precontraction}}\times100\%.$$

Concentration–response curves for ACh and SNP were constructed accordingly.

**Simulation analysis of Interacting Proteins**

Protein sequences for VDAC1 and HK2 were retrieved from the UniProt database (<https://www.uniprot.org/>)^3^. Structural models of protein‒protein interactions were predicted via alphafold3 (<https://alphafoldserver.com/>)^4^. Protein interaction interfaces were visualized and analyzed via PyMOL software ^5^.

**References**

1. Velsko IM, Chukkapalli SS, Rivera MF, et al. Active Invasion of Oral and Aortic Tissues by Porphyromonas gingivalis in Mice Causally Links Periodontitis and Atherosclerosis. Glogauer M, ed. *PLoS ONE*. 2014;9(5):e97811. doi:10.1371/journal.pone.0097811

2. Velsko IM, Chukkapalli SS, Rivera-Kweh MF, et al. Periodontal Pathogens Invade Gingiva and Aortic Adventitia and Elicit Inflammasome Activation in αvβ6 Integrin-Deficient Mice. Blanke SR, ed. *Infect Immun*. 2015;83(12):4582-4593. doi:10.1128/IAI.01077-15

3. The UniProt Consortium, Bateman A, Martin MJ, et al. UniProt: the Universal Protein Knowledgebase in 2023. *Nucleic Acids Res*. 2023;51(D1):D523-D531. doi:10.1093/nar/gkac1052

4. Abramson J, Adler J, Dunger J, et al. Accurate structure prediction of biomolecular interactions with AlphaFold 3. *Nature*. 2024;630(8016):493-500. doi:10.1038/s41586-024-07487-w

5. Rosignoli S, Paiardini A. Boosting the Full Potential of PyMOL with Structural Biology Plugins. *Biomolecules*. 2022;12(12):1764. doi:10.3390/biom12121764

1. **Supplementary Figures**


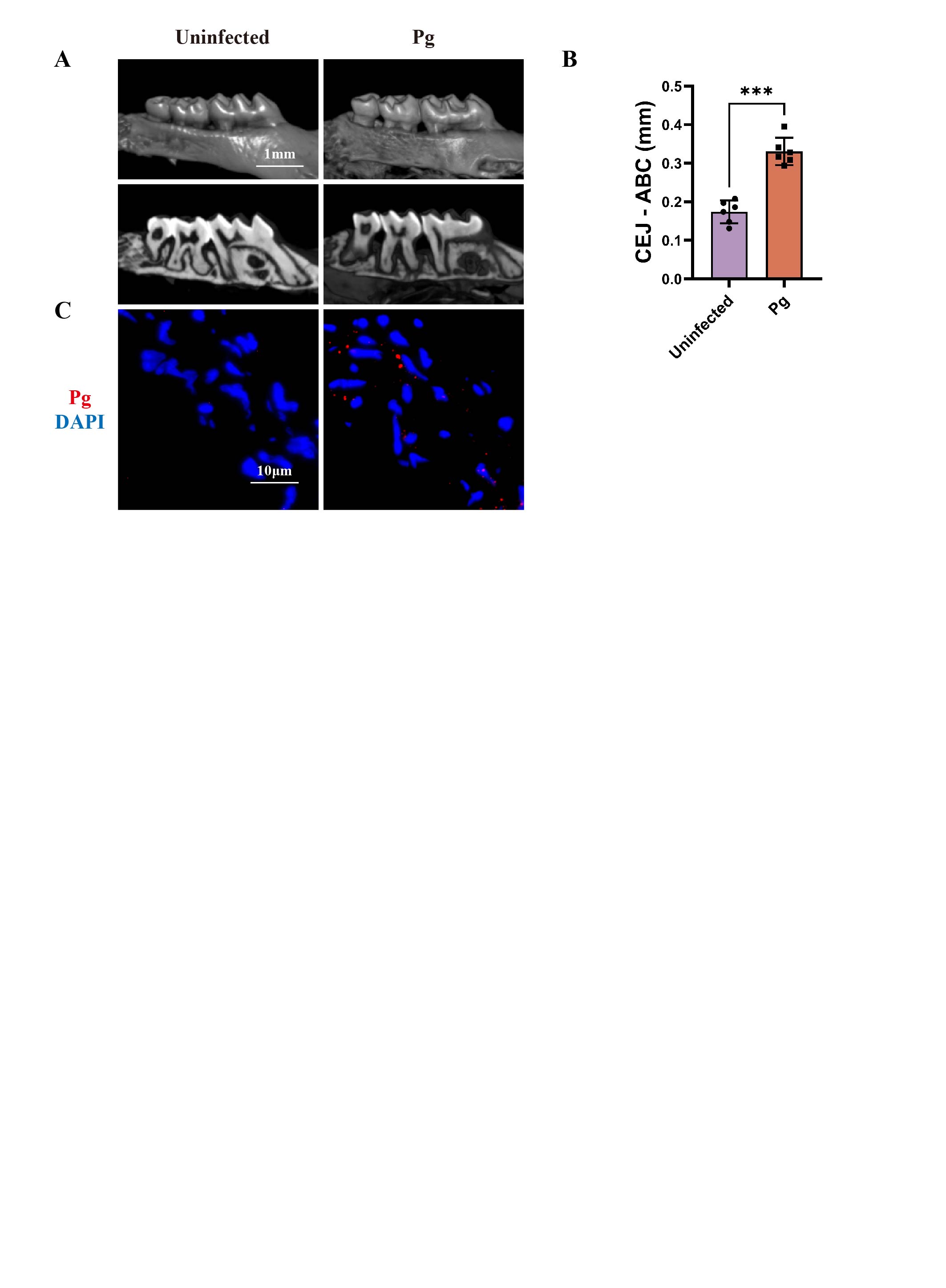


**Suppl Figure S1.** Oral application of *P. gingivalis* induced localized alveolar bone resorption and translocated to the aorta. (A, B) Representative micro–computed tomography images of maxillae in the 2 groups of mice. Scale bar = 1 mm. Measurement of alveolar bone resorption using distance from the CEJ to ABC of the mesial side of second molars. (n = 6). (C) FISH illustrating *P. gingivalis* (red) in *P. gingivalis group* mice aortic vessel. Scale bar = 10 mm. (n = 6). Pg, *P. gingivalis*. All numbers (n) are biologically independent experiments. ****P* < 0.001.


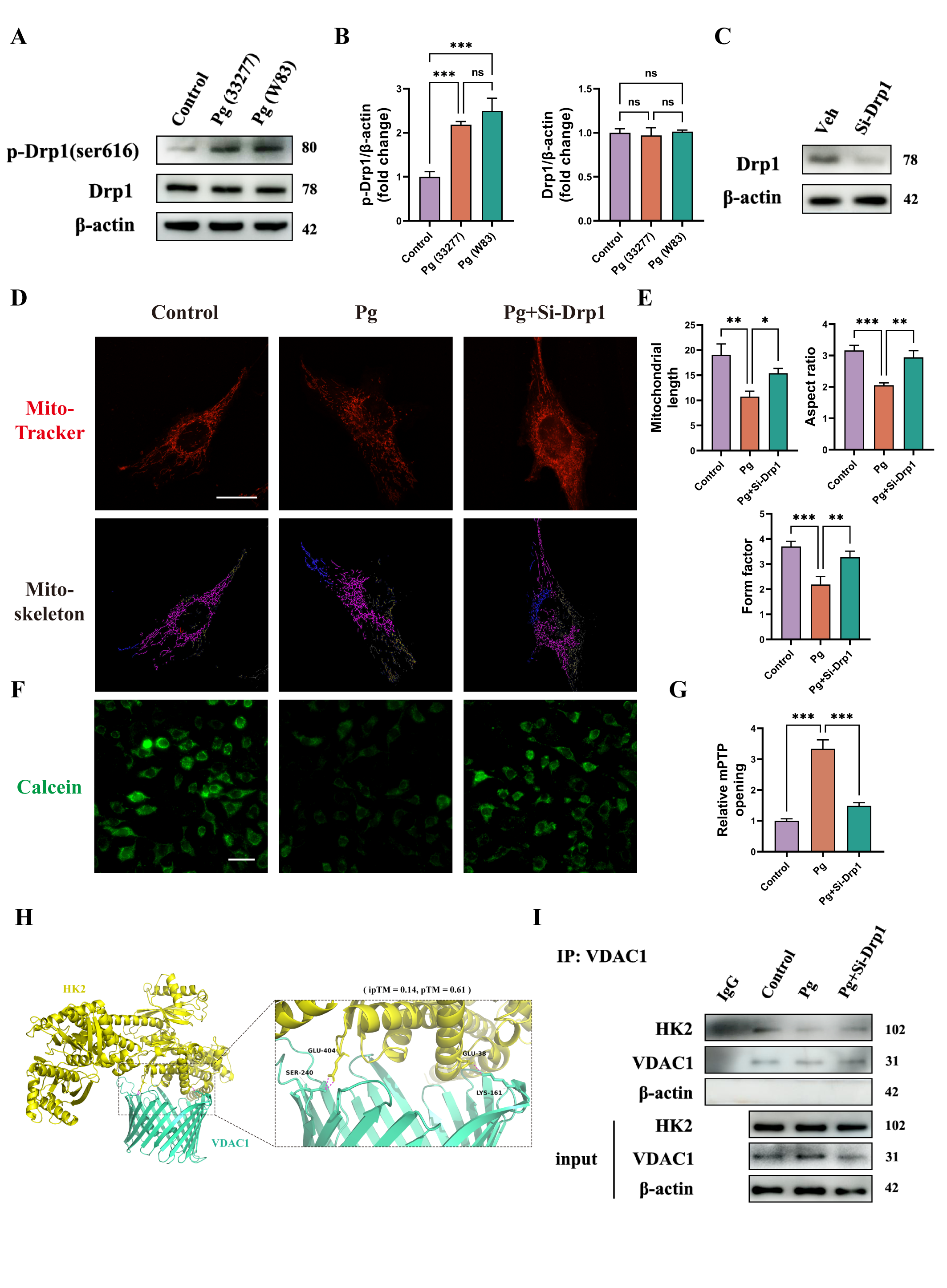


**Suppl Figure S2.** Drp1 knockdown attenuates *P. gingivalis*-induced mitochondrial fragmentation, mPTP opening and restores the VDAC1-HK2 association. (A, B) Both *P. gingivalis* strains 33277 and W83 increased Drp1 phosphorylation(ser616) in HAECs. HAECs were infected by *P. gingivalis* 33277 or W83 strain for 24 h (MOI = 100). p-Drp1(ser616) and Drp1 protein levels were measured in HAECs. (n = 3). (C) Drp1 protein levels were measured in HAECs transfected with either vehicle or Si-Drp1. (n = 3). (D, E) Representative confocal images of mitochondrial morphology in P. gingivalis-infected HAECs (MOI = 100 for 24h) following transfected with Si-Drp1. Mitochondrial length in each group was calculated by Image J. Scale bars =10 μm. (n = 3). (F, G) Calcein staining assay and quantitative bar chart. Scale bars = 20 μm. (n = 3). HAECs was infected with P. gingivalis (MOI = 100 for 24h) following transfected with Si-Drp1. (H) The modelled structure of VDAC1 and HK2 proteins. The main residues of VDAC1 and HK2 involved in the interaction are shown. (I) The interaction of VDAC1 and HK2 was validated using Co-immunoprecipitation assays. (n = 3). Pg, *P. gingivalis*. All numbers (n) are biologically independent experiments. ns = not significant. ****P* < 0.001.


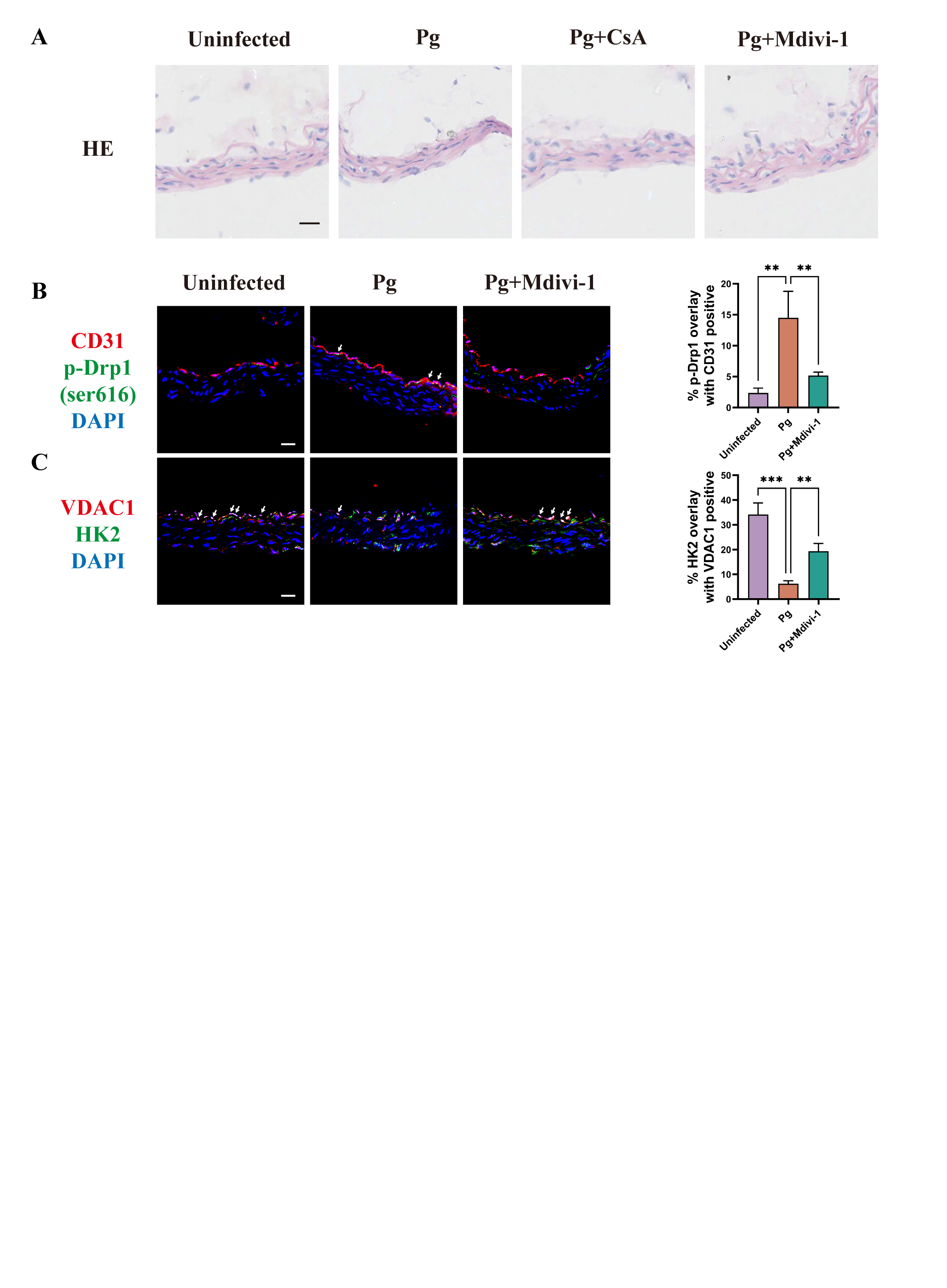


**Suppl Figure S3.** *P. gingivalis* induces Drp1 activation and HK2 dissociation in the aortic endothelium in vivo. (A) Representative HE staining images showing the structural integrity of the aortic wall. scale bars = 20 μm. (n = 6). (B) Immunofluorescence staining showing the endothelial expression of p-Drp1 (Ser616) and its co-localization with CD31. White arrows indicate regions of co-localization. scale bars = 20 μm. (n = 6). (C) Representative images showing the co-localization of VDAC1 and HK2 in the aortic endothelial layer. White arrows indicate regions of co-localization. scale bars = 20 μm. (n = 6). Pg, *P. gingivalis*. All numbers (n) are biologically independent experiments. ***P* < 0.01. ****P* < 0.001.

1. **Supplementary Table**

**Supplementary Table S1**

| **Antibody** | **Source** | **Dilution** |
| --- | --- | --- |
| Drp1 | Proteintech, China | 1:4000 (WB) |
| p-Drp1(ser616) | Cell Signaling Technology, USA | 1:1000 (WB), 1:400 (IF) |
| Mfn1 | Proteintech, China | 1:5000 (WB) |
| OPA1 | Proteintech, China | 1:2000 (WB) |
| Fis1 | Proteintech, China | 1:2000 (WB) |
| Cox IV | Proteintech, China | 1:6000 (WB) |
| CytC | Proteintech, China | 1:1000 (WB) |
| VDAC1 | Proteintech, China | 1:5000 (WB) |
| VDAC1 | Abcam,UK | 1:400 (WB, IF) |
| HK2 | Proteintech, China | 1:3000 (WB), 1:500 (IF) |
| β-actin | Proteintech, China | 1:6000 (WB) |
| CD31 | Servicebio, China | 1:1000 (IF) |

WB, Western blot; IF, immunofluoresence
